# Development of TiO_2_/ZrO_2_ Multi-Material Obtained from Ceramic Pastes for Material Extrusion

**DOI:** 10.3390/mi14122177

**Published:** 2023-11-29

**Authors:** Arseniy Repnin, Anton Sotov, Anatoliy Popovich, Dmitriy Masaylo

**Affiliations:** Institute of Machinery, Materials, and Transport, Peter the Great St. Petersburg Polytechnic University (SPbPU), Polytechnicheskaya, 29, 195251 Saint Petersburg, Russia

**Keywords:** additive manufacturing, material extrusion, ceramic materials, multi-material, TiO_2_/ZrO_2_

## Abstract

The application of additive manufacturing method such as material extrusion (MEX) allows the successful fabrication of ceramic products, including multi-ceramic products. Promising materials in this research area are TiO_2_ and ZrO_2_ ceramics, which can be used in electrical and electronic engineering. The aim of this work is to investigate the possibility of fabricating TiO_2_/ZrO_2_ multi-materials from ceramic pastes that can be used in the MEX. In this work, defects, chemical and phase composition, and microhardness were analyzed in multi-ceramic samples after sintering. Multi-ceramic TiO_2_/ZrO_2_ samples after the sintering process without interlayer could not be fabricated due to a too large difference in shrinkage between TiO_2_ and ZrO_2_. The samples with one and three interlayers also have defects, but they are less significant and can be fabricated. The average hardness for the TiO_2_ zone was 636.7 HV and for the ZrO_2_ zone was 1101 HV. In the TiO_2_ zone, only TiO_2_ phase in rutile is observed, while in the interlayer zones, in addition to rutile, ZrO_2_ and ZrTiO_4_ are also present, as is a small amount of Y_2_O_3_. In the zone ZrO_2_, only the ZrO_2_ phase is observed. The chemical analysis revealed that the interlayers comprise sintered ZrO_2_ granules enveloped by TiO_2_, ZrO_2_, and ZrTiO_4_.

## 1. Introduction

Unlike subtractive manufacturing, where material is removed, additive manufacturing (AM) enables the production of precise 3D geometry by adding material layer by layer based on a 3D model [1]. AM allows the use of different materials, including polymers, metals, ceramics, glasses, biomaterials, and composites, and encompasses several types of methods, such as stereolithography, selective laser melting, direct energy deposition, material extrusion, material jetting, etc. [2]. The technological advantages of AM allow us to obtain ceramic materials of complex geometries that are challenging or impossible to manufacture through traditional manufacturing [2]. There are several ways to produce ceramic materials via AM methods, such as binder jetting [3], vat photopolymerization (VT) [4], material jetting, and material extrusion [5]. It should be noted that the production of ceramic materials using these methods has certain technological limitations. The additional process of burning complex polymers and preparing suspensions (filament) based on them complicates and increases the production time [6]. There has been recent active research into producing ceramic and metal products using pastes via material extrusion (MEX); this process is also known as Direct Ink Writing (DIW) [7], Robocasting [8], or Metal Paste Deposition (MPD) [9]. This technological process is unrestricted by the aforementioned limitations. The advantages of MEX include material adjustment, an ability to adapt to various product geometries, and the use of a broad range of materials.

The commonly utilized ceramic materials presently in MEX are Titania (TiO_2_), Zirconia (ZrO_2_), Silicon Carbide (SiC), and Alumina (Al_2_O_3_). Titania is applied in bone and tissue engineering [10], Zirconia in biomedical engineering [11], Silicon Carbide in electronics and insulation [12], and Alumina in refractory tasks [13]. Titania possesses characteristics such as high dielectric constant and high fracture strength [14]. The material exhibits good bioactivity and biocompatibility, making it appealing for use in implants [15]. Titania possesses excellent catalytic and semiconducting properties [16]. Moreover, this ceramic is a commonly applied photocatalyst and greatly enhances the degradation efficiency of contaminants [17]. Zirconia exhibits high fracture toughness and bending strength and has thermal and chemical stability, as well as good ionic conductivity [18]. Moreover, Zirconia possesses excellent corrosion resistance, making it a favored material in the industrial and medical sectors. Yttrium-stabilized zirconium oxide is commonly used for product fabrication [19]. Zirconia finds applications in various fields, such as resistive heating, cutting tools, oxygen sensors, pump impellers, surgical implants, etc. [20].

Recently, research into the creation of multi-materials via AM methods has been of great interest [21,22,23]. Products that have multi-material structures possess enhanced performance [24] and are utilized in various fields, including the automotive industry, aerospace engineering, biomedicine, and the defense industry [25,26]. The MEX can be employed to produce ceramic multi-materials [27,28]. This technology does not have some limitations that are inherent to other AM methods of producing ceramic multi-materials [29]. For example, other methods may have problems with changing the chemical composition of the material within a single layer (for binder jetting and VT). Another advantage of using MEX over VT is the wider variety of materials available for MEX printing, since there are no restrictions on the optical properties of the materials. Additionally, MEX pastes have higher viscosity, allowing more ceramic powder to be added to the paste, thus resulting in the production of products with higher densities. Chao Xu et al. [30] investigated samples that were fabricated from pastes containing steel, copper, and Al_2_O_3_. The study aimed to explore the interactions between dissimilar metals and ceramics. The results showed no significant differences in volume shrinkage and no increased porosity in the interfacial zone. However, no alloy and ceramics mixing were detected in the system. The multi-material system of steel with Al_2_O_3_ exhibited a lower Young’s modulus, which was 17% lower than that of a pure steel sample. Pelz et al. [31] utilized the MEX to manufacture functional gradient material from boron carbide (B_4_C) and Silicon Carbide (SiC). The fabrication of this gradient material was achieved by mixing two pastes with different compositions. Additionally, various ceramic layered samples were made. These samples exhibit cracks induced through residual stresses caused by thermodynamic factors. Eric Schwarzer-Fischer et al. [32] examined a ZrO_2_/TiO_2_ multi-ceramic for electrical and electronic engineering, in which TiO_2_ and ZrO_2_ were separately produced via VT and were co-sintered afterwards. Both Titania and Zirconia exhibit comparable sintering behavior and can be sintered under identical conditions. After sintering, the homogeneous texture of the samples of both materials was achieved. Locally, there is a slight gap between the ceramics caused by differences in shrinkage and the coefficient of thermal expansion.

Additive manufacturing offers new possibilities in design by creating multi-material structures in products, which enhances their performance. MEX technology enables the successful production of ceramic and multi-ceramic products. Promising materials produced via MEX technology include TiO_2_ and ZrO_2_, which can be used in for electrical and electronic engineering [32]. Research has been conducted into the production of these ceramics using MEX, and multi-material fabrication via VT was carried out. Studies of the multi-material fabrication of these ceramics via MEX have not been carried out. In this regard, the aim of this work is to investigate the possibility of the fabrication of TiO_2_/ZrO_2_ multi-materials from ceramic pastes that can be used in MEX. To achieve the aim, the following tasks must be completed: determining the influence of ceramic pastes made from TiO_2_ and ZrO_2_, as well as their mixture in transition layers on the formation of multi-ceramic samples, and examining the defects, chemical and phase composition, and micro-hardness of multi-ceramic samples after sintering.

## 2. Materials and Methods

### 2.1. Starting Materials and Paste Preparation

**Figure 1:**
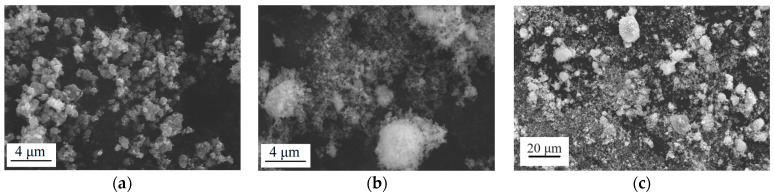
Morphology of ceramic powders: (**a**) powder of TiO_2_; (**b**) powder of ZrO_2_; (**c**) powder of 30% TiO_2_ и 70% ZrO_2_ (3-IL).

To obtain multi-material TiO_2_/ZrO_2_ samples, TiO_2_ and ZrO_2_ (partially stabilized with 3 mol.-% yttria, Tosoh Bioscience A.G., Griesheim, Germany) ceramic powders were used (Figure 1a,b). TiO_2_ powder has a particle size D(50) of 0.3 μm, while the ZrO_2_ powder has a granule size D(50) of 60 μm and particle size D(50) of 0.6 μm. Since there is a significant difference in particle size distribution between the two ceramic powders, ZrO_2_ powder was mechanically milled to create a ceramic paste. This process allowed the partial pulverization of the ZrO_2_ granules on their constituent particles. The reduction in granule size should lead to a more uniform shrinkage of future TiO_2_/ZrO_2_ multi-ceramic samples. Powder mixtures for interlayers with different mass ratios of TiO_2_ and ZrO_2_ were obtained: 70% TiO_2_ and 30% ZrO_2_ (Interlayer № 1, 1-IL); 50% TiO_2_ and и 50% ZrO_2_ (Interlayer № 2, 2-IL); and 30% TiO_2_ and 70% ZrO_2_ (Interlayer № 3, 3-IL). The requirement of the ceramic amount for interlayers arises from the varying shrinkage values of TiO_2_ and ZrO_2_. To mitigate this effect, the selection of powder concentrations within the layers was based on a gradient change in the ceramic amount in multi-material TiO_2_/ZrO_2_ samples: 0–50–100 wt.% of ZrO_2_ and 0–30–50–70–100 wt.% of ZrO_2_.

Figure 1 illustrates that TiO_2_ powder comprises irregularly shaped particles that are agglomerated (Figure 1a). The grinding of the ZrO_2_ powder did not result in the complete conversion of all granules into particles (Figure 1b). Figure 1c shows the morphology of 3-IL as the most representative example of the powder mixture composition. There, ZrO_2_ particles are represented as granules (lighter in color), and the mixture of finer particles consists of both TiO_2_ and ZrO_2_ particles (it is not possible to distinguish between TiO_2_ and ZrO_2_ particles in the mixture based on color). The presence of larger ZrO_2_ granules can lead to the non-uniform shrinkage of multi-ceramic samples and the non-uniform distribution of chemical composition in the zones of powder mixtures. A 4% aqueous solution of polyvinyl alcohol (PVA) was utilized as a binder for ceramic paste made from TiO_2_ and ZrO_2_, as well as from powder mixtures. To prepare the solution, PVA’s crystals were constantly stirred in water at 80 °C until completely dissolved, which took about 2 h.

### 2.2. MEX 3D-Printer

**Figure 2:**
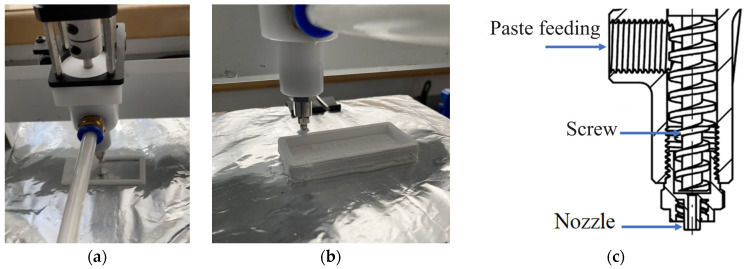
Material extrusion 3D-Printer—Tronxy Moore 1 Mini Clay: (**a**,**b**) printing of TiO_2_ paste; (**c**) paste printing implementation scheme.

The Tronxy Moore 1 Mini Clay 3D printer (Shenzhen Tronxy Technology Co., Ltd., Shenzhen, China, Figure 2a,b) was selected for the potential printing of prepared ceramic paste. This printer used an extrusion method to print the ceramic paste, which was facilitated using a printhead comprising a screw and nozzle (Figure 2c). The printer can be adapted to print multiple materials using two printheads or utilizing two containers of ceramic paste. Ceramic paste was formed layer by layer to create multi-material samples both without and with interlayers. The printing parameters for TiO_2_ paste, which, with some modifications, can be used for printing ZrO_2_ paste or for printing multi-material samples, were as follows: layer height—1.5 mm; nozzle diameter—1.5 mm; nozzle speed—30 mm/s; and flow—100%. Subsequent works will investigate how the technological process affects the structure and properties of multi-ceramic samples.

Various paste compositions were examined to study how the amount of ceramics in the paste affects linear shrinkage. Indeed, the TiO_2_ concentration was 46, 48, 50, and 52 wt.% of the ceramic powder; for ZrO_2_, it was 68, 70, 72, and 74 wt.% of the ceramic powder. The compositions were determined to provide the possibility of printability on a Tronxy Moore 1 Mini Clay 3D-Printer. Simplified tests of the considered paste were carried out, for which the possibility of printability was investigated. The designated ranges of the amount of ceramics guarantee satisfactory processability.

### 2.3. Multi-Ceramic Samples and Post-Treatment

**Figure 3:**
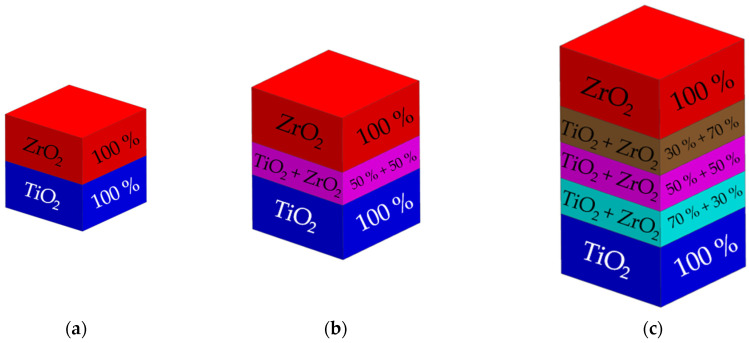
Multi-ceramic samples: (**a**) multi-ceramic sample without interlayer (MCS0); (**b**) multi-ceramic sample with one interlayer (MCS1); (**c**) multi-ceramic sample with three interlayers (MCS3).

Three different types of multi-material TiO_2_/ZrO_2_ samples were considered: the MCS0—multi-ceramic samples without a interlayer (which contained 100 wt.% TiO_2_ and 100 wt.% ZrO_2_); the MCS1—multi-ceramic samples with one interlayer: 2-IL; and the MCS3—multi-ceramic samples with three interlayers: 1-IL, 2-IL, and 3-IL (Figure 3). Several options for pastes for TiO_2_ and ZrO_2_ ceramics with different mass contents of powder were considered. The pastes from the powder mixtures had the following content of ceramic powder: 1-IL—55 wt.%; 2-IL—60 wt.%; and 3-IL—65 wt.%. The multi-material TiO_2_/ZrO_2_ samples from ceramic pastes were sintered in a muffle furnace using the following regime: heating up to 240 °C at a rate of 10 °C/min, before holding for 1 h; heating up to 500 °C at a rate of 5 °C/min, before holding for 1 h; and heating up to 1400 °C at a rate of 10 °C/min, before holding for 2 h.

### 2.4. Characterizations

The samples after sintering were examined using the Leica M125 stereomicroscope and the Leica DMi8 M optical microscope (Leica Microsystems, Wetzlar, Germany). Microhardness was measured using the Vickers MicroMet 5101 microhardness tester (Buehler Ltd., Lake Bluff, IL, USA). The chemical composition was studied using the scanning electron microscope Mira 3 (TESCAN, Brno, Czech Republic), equipped with an energy dispersive X-ray spectroscopy module. The phase composition was evaluated via X-ray microdiffraction with a beam width of 100 µm using a Rigaku SmartLab diffractometer (CuKα radiation, Rigaku Corporation, Tokyo, Japan).

## 3. Results and Discussion

### 3.1. The Linear Shrinkage Study and the Characterization of Multi-Ceramic Samples after Sintering

**Figure 4:**
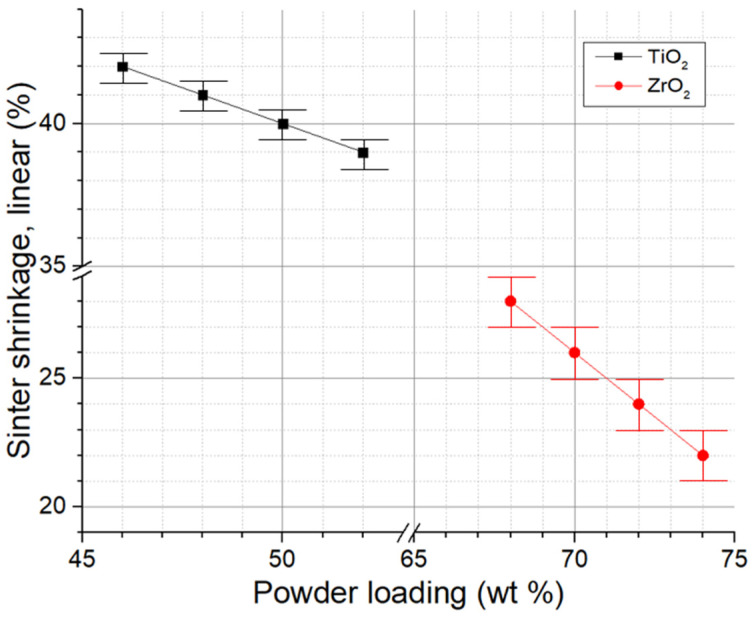
Sintering shrinkage in x- and y-directions of TiO_2_ and ZrO_2_ ceramics depending on the solid content.

Based on information about the particle size distribution of TiO_2_ and ZrO_2_ powders and analysis of the results of the powder morphology (Section 2.1), it can be concluded that non-uniform linear shrinkage is possible after the sintering of multi-ceramic samples. Figure 4 depicts the outcomes of the linear shrinkage alteration in TiO_2_ and ZrO_2_ samples after sintering, based on the contents of ceramic powder in the pastes. Linear shrinkage for TiO_2_ ranges from 39 to 42%, while for ZrO_2_, it ranges from 22 to 28%. A linear relation can be observed in the variation in linear shrinkage based on the ceramic particle content in the paste, both for TiO_2_ and ZrO_2_ powders. For the last one, the shrinkage change is more pronounced. The findings reveal distinctions in linear shrinkage for different ceramics that were previously explained. Uneven shrinkage can lead to the formation of defects in the fabrication of multi-ceramic materials. To minimize this effect, it is important to select paste compositions with similar shrinkage values. For future investigations, the selected compositions comprised 50 wt.% TiO_2_ powder and 70 wt.% ZrO_2_ powder, which led to a 14% variation in the linear shrinkage. Secondly, it is worth considering the formation of interlayers in multi-ceramic samples. These layers should be close in linear shrinkage values to both TiO_2_ and ZrO_2_, as well as to each other.

Three different types of powder mixtures, namely 1-IL, 2-IL, and 3-IL, were considered to produce interlayers. The linear shrinkage for 1-IL was 35%, while it was 32% and 30% for 2-IL and 3-IL, respectively. It can be seen that the presence of interlayers can reduce the difference in linear shrinkage. In the MCS1 the linear shrinkage discrepancy between TiO_2_ and the 2-IL will reach about 8%, as well as about 6% between ZrO_2_ and the 2-IL. In the MCS3, the linear shrinkage deviation between TiO_2_ and the 1-IL will be about 6%, while between 1-IL and 2-IL, it is 3%; between 2-IL and 3-IL, it is 2%; and between 3-IL and ZrO_2_, it is approximately 4%. It should be noted that the researchers who carried out a study of TiO_2_/ZrO_2_ multi-ceramic samples utilizing the VP method determined a shrinkage difference of less than 1% [32]. Achieving this value using prepared ceramic pastes is not feasible, but the difference from the literature remains insignificant.

**Figure 5:**
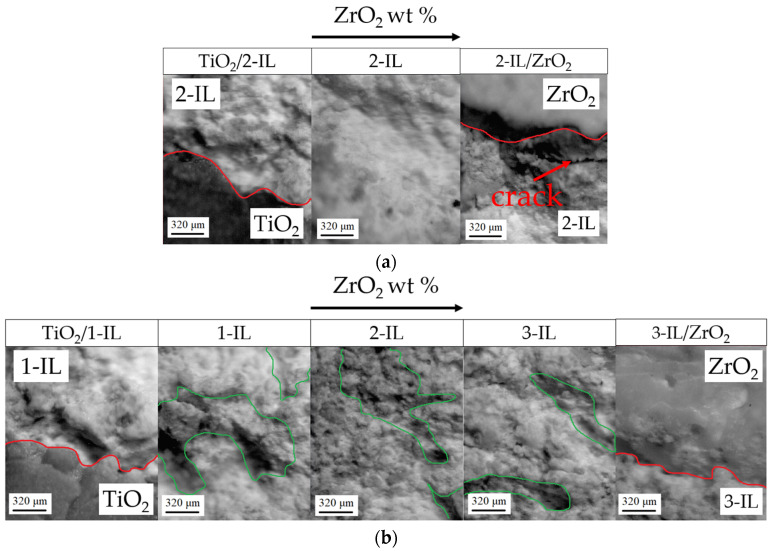
Multi-ceramic samples after sintering: (**a**) the MCS1; (**b**) the MCS3; red line—interface between zones with different compositions; green line—boundaries of pitting zones.

After sintering the multi-ceramic samples, it was found that the MCS0 had significant defects due to a too large difference in shrinkage and cannot be fabricated (this sample was not further investigated). On the other hand, the MCS1 and MCS3 samples exhibited fewer defects, and fabrication was feasible. It should be noted that partial sintering occurs in the interlayers. In this regard, there is no possibility of analyzing the interlayers using an optical microscope, and the studies were carried out using a stereomicroscope. Figure 5a illustrates that defects, specifically cracks, are present at the interface between TiO_2_ and ZrO_2_ ceramics and the 2-IL in the MCS1. These defects could be due to non-uniform shrinkage. Consequently, it can be concluded that using only 2-IL is insufficient for reducing differences in shrinkage, and it is more suitable to employ three interlayers, i.e., 1-IL + 2-IL + 3-IL. So, no cracks were observed at the interfaces in the MCS3. Interactions at the interface between the interlayers may cause alterations in concentration. An extensive investigation of the interface is required to study this phenomenon. It can be seen from Figure 5b that as the ZrO_2_ content increases, the degree of sinterability in the interlayers increases. This can be qualitative observed by the amount of material that is pitting (Figure 5b). This phenomenon can be explained by the fact that ZrO_2_ in 1-IL is unevenly distributed (presence of granules and particles) and presented in low quantities. Consequently, the ceramic materials that are not sintered with each other tend toward pitting. In comparison, 3-IL exhibits larger quantities of sintered ZrO_2_ and lower amounts of evenly distributed TiO_2_, which began to exhibit pitting.

### 3.2. The Defect Analysis and the Hardness Study of Multi-Ceramic Samples after Sintering

The TiO_2_ and ZrO_2_ zones in MCS3 were analyzed for the presence of defects using an optical microscope. In the TiO_2_ zone, a small number of pores and cracks occur, while in the ZrO_2_ zone, though pores also occur, no cracks are present, but there are branched crack-like defects [33]. Pores may occur due to the presence of air bubbles in the paste, but their number can be reduced by degassing the paste (this step was not carried out in this work). Cracks may occur due to the insufficient amount of powder in the paste. Therefore, it is preferable to increase the TiO_2_ powder proportion to reduce the probability of crack formation. Branched crack-like defects develop at the boundaries of powder granules and are dependent on the granule’s size and shape, binder properties, and binder removal process [34].

**Figure 6:**
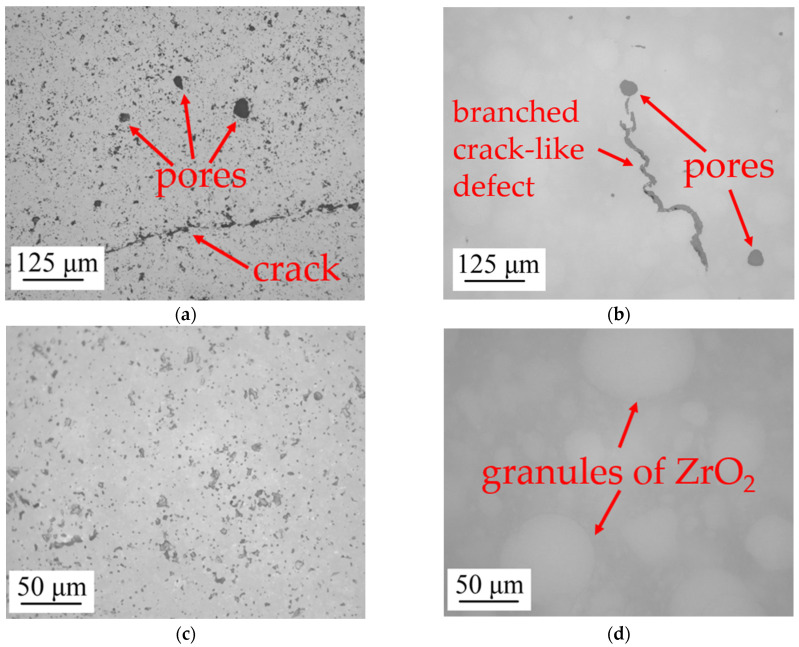
Optical microscope images in the TiO_2_ and ZrO_2_ zones of the MCS3: (**a**) defect analysis of the TiO_2_; (**b**) defect analysis of the ZrO_2_; (**c**) study of the TiO_2_ structure; (**d**) study of the ZrO_2_ structure.

Upon closer examination of the TiO_2_ and ZrO_2_ zones, it can be seen that the zone of TiO_2_ has porosity with a pore size of 10 microns. This porosity could be explained by the presence of voids among the agglomerations of TiO_2_ particles. It is possible that these voids contained a binder that evaporated during removal. Another possible explanation is that densification in TiO_2_ was not completely achieved, resulting in residual porosity. Using ImageJ software, the porosity in Figure 6c was quantified, and the value obtained was 8.83%. In the zone of ZrO_2_, the sintered granules of this ceramic, which were not milled and were sintered using the surrounding milled ZrO_2_ particles, were observed.

The microhardness results for the TiO_2_ and ZrO_2_ zones of the MCS3 are presented in Table 1. The TiO_2_ zone had an average hardness of 636.7 HV, which is below the literature data value of around 850 HV [35]. This could be attributable to the porous structure. To enhance hardness in the future, technological advancements can be made in paste preparation. The ZrO_2_ zone had an average hardness of 1101 HV, which is slightly lower than the literature value of approximately 1200 HV [36]. The observed decrease in hardness may be attributed to insufficient pressure being applied while sintering the samples. Our results reveal that complete sintering took place in the TiO_2_ and ZrO_2_ zones. Knowing that there is a relationship between hardness and strength, it can be assumed that the strength in the TiO_2_ and ZrO_2_ zones is likely to be high [37]. It is not possible to determine the hardness of the interlayers, as they have not been fully sintered.

### 3.3. The Phase and Chemical Composition Analysis of the Multi-Ceramic Samples after Sintering

**Figure 7:**
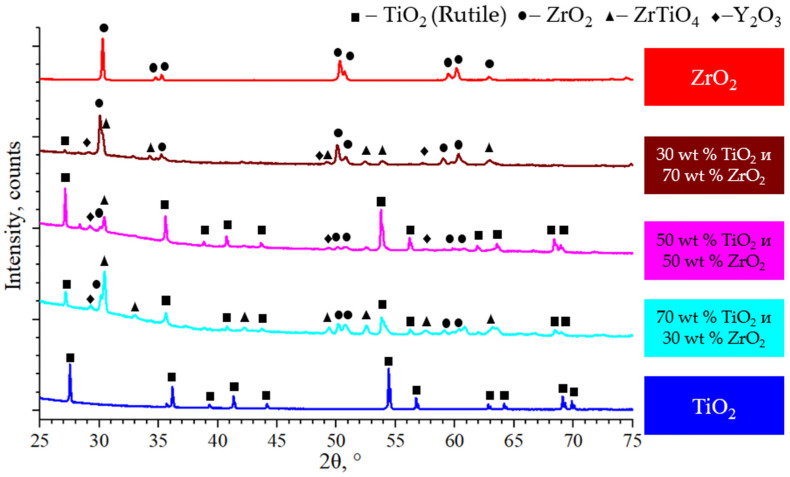
The phase composition of the MCS3.

Phase and chemical composition analyses were carried out for the MCS3 sample (Figure 7). Only TiO_2_ in the rutile phase was observed within the TiO_2_ zone, without any other phases being present. Conversely, in the 1-IL region, apart from rutile, ZrO_2_, ZrTiO_4_, and a small amount of Y_2_O_3_ were identified. As ZrO_2_ content increased, the quantities of ZrTiO_4_ and TiO_2_ decreased, while that of Y_2_O_3_ increased. The ZrTiO_4_ peaks’ intensities increased as the amount of TiO_2_ increased due to the lowering of the tetragonal–monoclinic transformation temperature, leading to the formation of solid solutions of TiO_2_ in ZrO_2_ [38]. The TiZrO_4_ phase is formed at 1700 °C, as per the TiO_2_-ZrO_2_ state diagram. However, earlier research indicated that the TiZrO_4_ phase remains stable above 1100 °C, resulting in the suggestion that TiO_2_ contributes toward stabilizing this phase [39]. A deviation in TiO_2_ peaks between different interlayers was observed. This suggests that there is a change in the lattice parameter of TiO_2_. It can be assumed that this phenomenon is related to the presence of Zr atoms as impurity in TiO_2_. In the ZrO_2_ zone, no peaks of TiO_2_ and ZrTiO_4_ phases were observed, as well as in the Y_2_O_3_ phase; the latter behaves like an alloying agent and cannot be detected via X-ray diffraction.

Using a scanning electron microscope equipped with an energy dispersive X-ray spectrometer, the chemical compositions of different zones of MCS3 were determined. In the TiO_2_ zone, elements such as Ti and O were observed, the ratios of which corresponded to the TiO_2_ ceramic (Table 2). In the ZrO_2_ zone, elements like Zr, O, and Y were observed, which corresponded to the ZrO_2_ ceramic stabilized by Y (Table 2). It can be noted that in the TiO_2_ zone, there were no elements from the ZrO_2_ zone and vice versa. This suggests the absence of material mixing between the different zones when forming multi-ceramic samples. This kind of feature is important, as it indicates that there is a possibility to carry out a controlled change in the chemical composition of the multi-ceramic sample.

**Figure 8:**
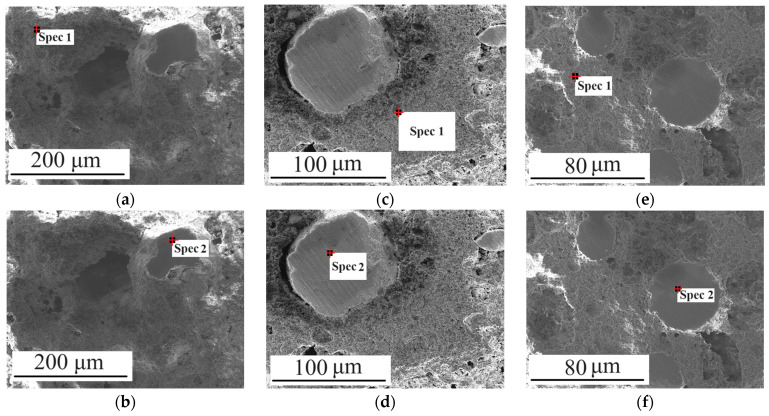
Study of chemical composition in the MCS3: (**a**,**b**) 1-IL; (**c**,**d**) 2-IL; and (**e**,**f**) 3-IL.

Based on the chemical composition and phase analysis of the interlayers, it appears that they consist of sintered granules of ZrO_2_, which are enveloped by TiO_2_, ZrO_2_, and ZrTiO_4_ that have not been completely sintered (Figure 8, Table 3). After sintering, powder mixtures of ZrO_2_ and TiO_2_ become ZrO_2_-ZrTiO_4_ composites as TiO_2_ reacts with ZrO_2_ and transforms into ZrTiO_4_ at temperatures above 1400 °C [40]. Table 3 illustrates a decrease in Ti content from 1-IL to 3-IL, which also indicates that there is a decrease in the amount of ZrTiO_4_ in ZrO_2_-ZrTiO_4_. This suggests that ZrO_2_ granules and ZrO_2_-ZrTiO_4_ are present, in addition to TiO_2_ particles, in 1-IL. In the 2-IL, sintered TiO_2_ particles, ZrTiO_4_ in ZrO_2_-ZrTiO_4_, and ZrO_2_ granules are present. However, there is a more noticeable decrease in the amount of ZrTiO_4_ in ZrO_2_-ZrTiO_4_ compared to TiO_2_ due to the reduced quantity of TiO_2_ and the non-uniform distribution of ZrO_2_. It can be assumed that since the increase in the amount of ZrO_2_ from 1-IL to 3-IL occurs not only due to particles but also due to granules, the amount of interaction between TiO_2_ particles and ZrO_2_ decreases. In this regard, it can be assumed that the quantity of interactions between TiO_2_ and ZrO_2_ particles decreases, meaning that ceramic particles start to interact more with their own kind. The presence of ZrO_2_ granules and the variation in chemical composition between particles leads to heterogeneity in the interlayer. However, this heterogeneity can be controlled within an interlayer, and according to the research conducted, it is unlikely to spread beyond the interlayers.

## 4. Conclusions

Additive manufacturing (AM) offers new possibilities in design by creating multi-material structures in products, which enhance their performance. The application of such an AM method as material extrusion (MEX) allows the successful fabrication of ceramic products, including multi-ceramic products. The aim of this work was to investigate the possibility of fabricating TiO_2_/ZrO_2_ multi-materials from ceramic pastes that can be used in MEX technology and electrical and electronic engineering. In this work, defects, chemical and phase composition, and microhardness were analyzed in multi-ceramic samples after sintering. The conducted research resulted in the following conclusions:

(1) Sintering the multi-ceramic samples without interlayer caused significant defects due to a too large difference in the shrinkage of TiO_2_ and ZrO_2_ pastes, and they cannot be fabricated. The samples with one and three interlayers exhibited fewer defects, and fabrication was feasible. However, partial sintering occurred in the interlayers, and in the sample with one interlayer, cracks appeared between the ceramics and interlayer. Consequently, it can be concluded that using only one interlayer is insufficient for reducing differences in shrinkage, and it is more suitable to employ three interlayers.
(2) In the TiO_2_ zone, a small number of pores and cracks occur, while in the ZrO_2_ zone, though pores also occur, no cracks are present, but there are branched crack-like defects. The zone of TiO_2_ has a porosity with a pore size of 10 microns, and in the zone of ZrO_2_, sintered granules of this ceramic with the surrounding ZrO_2_ particles were observed. The average hardness in the TiO_2_ zone is 636.7 HV, and in the ZrO_2_ zone, it is 1101 HV.
(3) Only TiO_2_ in the rutile phase is observed within the TiO_2_ zone, without any other phases being present. As ZrO_2_ content increased, the quantities of ZrTiO_4_ and TiO_2_ decreased, while that of Y_2_O_3_ increased. In the ZrO_2_ zone, no peaks of TiO_2_ and ZrTiO_4_ phases were observed, as well as the Y_2_O_3_ phase. Based on the chemical composition and phase analysis of interlayers, it appears that they contain sintered granules of ZrO_2_, which are enveloped by TiO_2_, ZrO_2_, and ZrTiO_4_ that have not been completely sintered.
(4) As a further line of research, firstly, we propose to carry out the adjusting of the print parameters for fabricating multi-ceramic samples from pastes via MEX. Secondly, we plan to evaluate the mechanical properties of multi-materials. Thirdly, we propose to analyze the electrical properties. Based on the obtained research results of this work, there is a possibility of considering other multi-ceramic systems.

## Figures and Tables

**Table 1:** The results of hardness studies in the TiO_2_ and ZrO_2_ zones of the MCS3.

Zone in the MCS3	Microhardness, HV	Average Microhardness, HV
TiO_2_	640.7	636.7
	635.5	
	630.8	
	637.4	
	639.1	
ZrO_2_	1120	1101.6
	1111	
	1080	
	1100	
	1097	

**Table 2:** Chemical composition in the TiO_2_ and ZrO_2_ zones of the MCS3.

Zone in Multi-Ceramic Sample	Elements	Average Content, wt.%
TiO_2_	Ti	59.95
	O	40.05
ZrO_2_	Zr	69.96
	O	25.71
	Y	4.33

**Table 3:** Study of chemical composition in the MCS3.

Interlayer	Zone form Figure 8	Elements	Content, wt.%
1-IL	a-Spec 1	Ti	18.24
		Zr	51.54
		O	30.25
	b-Spec 2	Zr	73.58
		O	26.42
2-IL	c-Spec 1	Ti	16.89
		Zr	53.18
		O	29.93
	d-Spec 2	Zr	74.03
		O	25.97
3-IL	e-Spec 1	Ti	6.64
		Zr	65.83
		O	27.53
	f-Spec 2	Zr	73.31
		O	26.69

## Data Availability

The main data used in this study are provided in this paper. Any other raw/processed data required to reproduce the findings of this study are available from the corresponding author upon request.
